# Sphingosine-1-Phosphate Is a Novel Regulator of Cystic Fibrosis Transmembrane Conductance Regulator (CFTR) Activity

**DOI:** 10.1371/journal.pone.0130313

**Published:** 2015-06-16

**Authors:** Firhan A. Malik, Anja Meissner, Illya Semenkov, Steven Molinski, Stan Pasyk, Saumel Ahmadi, Hai H. Bui, Christine E. Bear, Darcy Lidington, Steffen-Sebastian Bolz

**Affiliations:** 1 Department of Physiology, University of Toronto, Medical Science Building, 1 King’s College Circle, Toronto, M5S 1A8 Canada; 2 Department of Brain Ischemia and Neurodegeneration, Institut d'Investigacions Biomèdiques August Pi i Sunyer (IDIBAPS), Rosello 161, 6th floor, 08036 Barcelona, Spain; 3 Department of Biochemistry, University of Toronto, Medical Science Building, 1 King’s College Circle, Toronto, M5S 1A8 Canada; 4 Programme in Molecular Structure and Function in the Research Institute, The Hospital for Sick Children, 555 University Avenue, Toronto, MG5 1X8 Canada; 5 Lilly Research Laboratories, Indianapolis, Indiana 46285, United States of America; 6 Toronto Centre for Microvascular Medicine, University of Toronto and The Li Ka Shing Knowledge Institute at St. Michael’s Hospital, 209 Victoria Street, Toronto, M5B 1T8 Canada; 7 Heart & Stroke / Richard Lewar Centre of Excellence for Cardiovascular Research, University of Toronto, 50 College Street, Toronto, M5S 3E2 Canada; University of Pittsburgh, School of Medicine, UNITED STATES

## Abstract

The cystic fibrosis transmembrane conductance regulator (CFTR) attenuates sphingosine-1-phosphate (S1P) signaling in resistance arteries and has emerged as a prominent regulator of myogenic vasoconstriction. This investigation demonstrates that S1P inhibits CFTR activity via adenosine monophosphate-activated kinase (AMPK), establishing a potential feedback link. In Baby Hamster Kidney (BHK) cells expressing wild-type human CFTR, S1P (1μmol/L) attenuates forskolin-stimulated, CFTR-dependent iodide efflux. S1P’s inhibitory effect is rapid (within 30 seconds), transient and correlates with CFTR serine residue 737 (S737) phosphorylation. Both S1P receptor antagonism (4μmol/L VPC 23019) and AMPK inhibition (80μmol/L Compound C or AMPK siRNA) attenuate S1P-stimluated (i) AMPK phosphorylation, (ii) CFTR S737 phosphorylation and (iii) CFTR activity inhibition. In BHK cells expressing the ΔF508 CFTR mutant (CFTR^ΔF508^), the most common mutation causing cystic fibrosis, both S1P receptor antagonism and AMPK inhibition enhance CFTR activity, without instigating discernable correction. In summary, we demonstrate that S1P/AMPK signaling transiently attenuates CFTR activity. Since our previous work positions CFTR as a negative S1P signaling regulator, this signaling link may positively reinforce S1P signals. This discovery has clinical ramifications for the treatment of disease states associated with enhanced S1P signaling and/or deficient CFTR activity (e.g. cystic fibrosis, heart failure). S1P receptor/AMPK inhibition could synergistically enhance the efficacy of therapeutic strategies aiming to correct aberrant CFTR trafficking.

## Introduction

Sphingosine-1-phosphate (S1P) is a key endogenous regulator of resistance artery myogenic vasoconstriction [1–3]. Pressure elevation stimulates sphingosine kinase 1 (Sphk1) and hence, S1P production in microvascular smooth muscle cells (VSMCs) [2], which subsequently activates an array of pro-constrictive signaling cascades [4]. Specifically, S1P signaling concurrently activates myosin light chain kinase and inhibits myosin light chain phosphatase, thereby driving substantial myosin light chain phosphorylation and consequently, potent vasoconstriction. To finely tune S1P signaling, a robust degradation mechanism counterbalances endogenous S1P production. This mechanism depends on two key elements: first, the cystic fibrosis transmembrane conductance regulator (CFTR) transports extracellular S1P across the plasma membrane, thereby sequestering it from its receptors; the internalized S1P is then degraded by the intracellular S1P phosphohydrolase 1 (SPP1) [5,6].

Our previous work in resistance arteries has characterized Sphk1 and CFTR/SPP1 as the principal counteracting signaling elements within a signaling framework that precisely controls S1P bioavailability and consequently, its pro-constrictive actions. In principle, inversely regulating this signaling tandem (i.e., decreasing S1P degradation when production increases and vice versa) would enable more efficient control of S1P signal onset, amplitude and duration. However, while we have defined a rapid mechanism that enhances S1P synthesis in response to transmural pressure elevation [2], a similarly rapid mechanism that depresses S1P degradation (i.e., regulates CFTR/SPP1) has yet to be discovered (thus far, only transcriptional mechanisms have been defined [6]). Of the CFTR/SPP1 pair, CFTR is ideally positioned to partner the dynamic modulation of S1P degradation with S1P synthesis, since it functions as the primary bottleneck governing S1P degradation in VSMCs [5,6]. We hypothesize that S1P signaling depresses CFTR channel activation, thereby functionally linking S1P synthesis and degradation. We propose that adenosine monophosphate-activated protein kinase (AMPK), both a S1P signaling target [7,8] and a negative CFTR regulator [9,10], serves as the critical intermediary.

Although AMPK is primarily considered an energy-sensing enzyme regulated by cellular AMP levels [11], non-metabolic phosphorylation mechanisms (i.e., via LKB1 and CaMKKβ) also invoke robust (>1000 fold) increases in AMPK activity [12,13]. In vascular endothelial cells, S1P activates the AMPK signaling stream via the S1P_1_/S1P_3_ receptor subtypes [7,8], a mechanism that is likely to be broadly applicable, given the ubiquitous nature of the two signaling pathways [11,14]. Activated AMPK attenuates CFTR channel gating by phosphorylating inhibitory phosphorylation sites (Ser^737^ and Ser^768^) within CFTR’s regulatory domain (R-domain) [9,10]. We hypothesize that S1P signals harness this signaling mechanism to reduce CFTR-dependent S1P transport.

Unfortunately, assessing the effect of S1P signals on S1P uptake is impractical, due to significant confounds. Specifically, labeled S1P (e.g., radio-labeled or FITC-labeled) is both intrinsically active (i.e., activates S1P receptors) and competes with unlabeled S1P for CFTR-dependent transport: this makes it impossible to discern between signaling- and competition-based transport inhibition. We therefore utilized iodide efflux to measure total CFTR activity (i.e., the product of surface expression and channel gating), a strategic maneuver that eliminates these confounds and permits testing the core hypothesis: that S1P signaling modulates CFTR activity. This investigation characterizes a rapid, non-transcriptional mechanism that putatively amplifies stimulated S1P signals by transiently suspending S1P degradation.

## Materials and Methods

### Animal Use

This investigation conforms to the *Guide for the Care and Use of Laboratory Animals* published by the NIH (Publication No. 85–23, revised 1996). All animal care and experimental protocols were approved by the Institutional Animal Care and Use Committee at the University of Toronto and were conducted in accordance with Canadian animal protection laws.

Wild-type mice (2–3 months of age; C57BL/6N) were purchased from Charles River Laboratories (Montreal, Canada). Mice homozygous for the ΔF508 CFTR mutation (CFTR^tm1EUR^) [15] and the complementary wild-type control littermates (both FVB/129 strain background; all 2–3 months of age) were obtained from an established colony at the *Hospital for Sick Children* (Toronto, Canada). Prior to tissue collection (e.g., mesenteric arteries, lung tissue), animals were fully anesthetized with isoflurane and humanely euthanized by decapitation. Mesenteric arteries were micro-dissected and lysed for western blotting, as previously described [16]. A subset of experiments used mice with heart failure, which was induced by a standard procedure of left anterior descending coronary artery ligation [16]. Mice were fully anesthetized during this surgical procedure (isoflurane) and were given an analgesic (buprenorphine) for 2 days post-surgery. A more detailed description of the procedure can be found in the S1 File.

### Cell culture

Naïve Baby Hamster Kidney (BHK) cells (purchased from American Type Culture Collection [ATCC]; Manassas, USA) were maintained in DMEM/F12 media containing 5% fetal bovine serum under standard culture conditions (37°C, 5%CO_2_). BHK cells stably expressing wild-type CFTR (CFTR^wt^) [17] or the ΔF508 CFTR mutant (CFTR^ΔF508^) [17] were maintained in medium supplemented with 500μmol/L methotrexate (which activates the CFTR transgene promoter).

Cells (at 40–50% confluency) were transfected with plasmid DNA (2μg DNA per 35mm dish) or siRNA (25nmol/L per 22mm dish) using FuGene 6 transfection reagent (Promega; Madison, USA), according to the manufacturer’s instructions. Cells were transfected for 48 hours, at which point cell confluency was approximately 95%. This investigation utilized plasmids encoding mutated CFTR proteins containing either a serine-to-alanine mutation at residue 737 (CFTR^S737A^) or a glycine to aspartic acid mutation at residue 551 (CFTR^G551D^); siRNA reagents (“On-Target plus” siRNA targeting the human AMPK α1 catalytic subunit [PRKAA1; cat# L-005027–00] and control, non-targeting siRNA [cat# D-001810-10-05]) were purchased from Dharmacon, Inc. (Lafayette, USA).

### Iodide Efflux Assessment

Iodide efflux measurements were conducted as previously described [18,19]. Briefly, cells were loaded with iodide by incubating them in a HEPES-based loading buffer containing 136mmol/L NaI for 1 hour at 37°C (with 5% CO_2_), followed by several washes with iodide-free buffer (NaNO_3_ substituted for NaI). Iodide efflux is minimal under basal/non-stimulated conditions (Fig A in S1 File): thus, as previously described [19], CFTR-dependent iodide efflux was stimulated via the activation of protein kinase A (PKA). Supernatant iodide levels were measured with a calibrated iodide-selective electrode (Lazar Research Laboratories; Los Angeles, USA).

Our initial experiments continuously measured iodide efflux in “real time” [18]. Confluent, iodide-loaded monolayers (equivalent to 2,355mm^2^ growing area) were scraped, pelleted, washed and re-suspended into 250μl of efflux buffer. Iodide efflux was stimulated with forskolin (FSK; 20μmol/L in 1% DMSO) and real-time iodide electrode traces were recorded with a Digidata 1320A data acquisition system and Clampex 8 software (Molecular Devices; Sunnyvale, USA). In terms of kinetics, efflux was evident within 30 seconds (post-stimulation), was reliably maximal after 60 seconds and remained maximal for at least 2 minutes (a representative tracing is shown in Panel A of Fig A in S1 File); for consistency, the efflux rate was calculated as the slope between 60–120 seconds. Since it is difficult to control cell losses during harvesting/re-suspension, cells were permeabilized (0.1% Triton X-100) at the conclusion of the experiment: this provided a “total loaded iodide” value that indirectly assessed the number of intact cells in the system. Importantly, if plasma membrane integrity was compromised during cell harvesting, the released iodide is removed by washes prior to re-suspension; compromised cells, therefore, do not contribute to the measured signal. As expected, no significant differences in total loaded iodide were observed across sample sets.

Cells exposed to transfection reagents yielded inconsistent results in real-time measurements, likely because membrane fragility increased and accentuated cell losses during harvesting. We successfully adapted the procedure to mitigate this issue and in the process, significantly augmented the signal amplitude. Confluent, iodide-loaded cell monolayers grown on 25mm coverslips (490mm^2^) were sequentially transferred through 8 separate volumes of 2mL efflux buffer for 1 minute each. The efflux buffer contained 10μmol/L FSK, 1mmol/L isobutylmethylxanthine and 100μmol/L cpt-cAMP (in 1% v/v DMSO), a cocktail that more potently activates PKA compared to FSK alone. Supernatant iodide levels (cell free) were determined (iodide electrode) for each 1-minute interval (Panel B of Fig A in S1 File); cumulative efflux was plotted to assess the response kinetic (Panel C of Fig A in S1 File). Consistent with real time measurements, efflux was evident within the first minute and was reliably maximal between 60–120 seconds (Panel C of Fig A in S1 File). The efflux rates for this approach, therefore, are calculated using the efflux level between 60–120 seconds post-stimulation (i.e., consistent time frame as real-time measures).

### FACS-based measurement of FITC-S1P uptake

As previously described [6], cell monolayers (treated or untreated) were incubated with 1μmol/L S1P-FITC for 60 minutes; the cells were then detached by trypsinization, washed twice with ice-cold PBS, filtered through a 35μm cell strainer and analyzed using the Becton-Dickinson FACS Canto operated by FACS DIVA version 6.1 software. Cells monolayers treated with non-labeled S1P served as background controls. The analysis procedure determined the mean fluorescence intensity (arbitrary units) of each cell population, which is a measure of uptake.

### Western Blotting

We utilized standard procedures for western blotting; specific methodological and reagent details are provided in the S1 File. This investigation primarily uses a custom-made antibody to detect CFTR serine 737 phosphorylation (designated “67D4”), a generous gift from Dr. David Thomas (McGill University, Montreal, Canada). Validation of the 67D4 antibody’s specificity and phospho-sensitivity is shown in Fig B in S1 File. Dr. John Riordan (University of North Carolina—Chapel Hill) provided phosphorylation sensitive (“570”) and insensitive (“596”) CFTR antibodies through the *Cystic Fibrosis Foundation Therapeutics* Antibody Distribution Program (http://www.cff.org/research): these antibodies were used in the validation process. All other antibodies used in this study are commercially available.

The 67D4 and 570 CFTR antibodies specifically recognize a non-phosphorylated CFTR epitope containing serine 737. Consequently, the loss of antibody binding indicates increased phosphorylation. Western blots display the mature, fully-glycosylated form of CFTR (Band C), which predominates in BHK cells expressing CFTR^wt^; Band C is widely accepted as the functional form at the cell surface.

### Statistics

All data are expressed as means ± SEM, where *n* is the number of independent experiments. A non-parametric Mann-Whitney test with computation of exact *p*-values was utilized for the comparison of two independent groups. For comparison of multiple independent groups, a non-parametric one-way ANOVA (Kruskal-Wallis) followed by a Mann-Whitney test with exact *p*-value computation as a post-hoc test for pair-wise comparisons was employed (since this was exploratory, we did not adjust for multiple comparisons). Differences were considered significant at error probabilities of *P*<0.05.

## Results

### S1P attenuates CFTR-dependent iodide efflux

**Fig 1:**
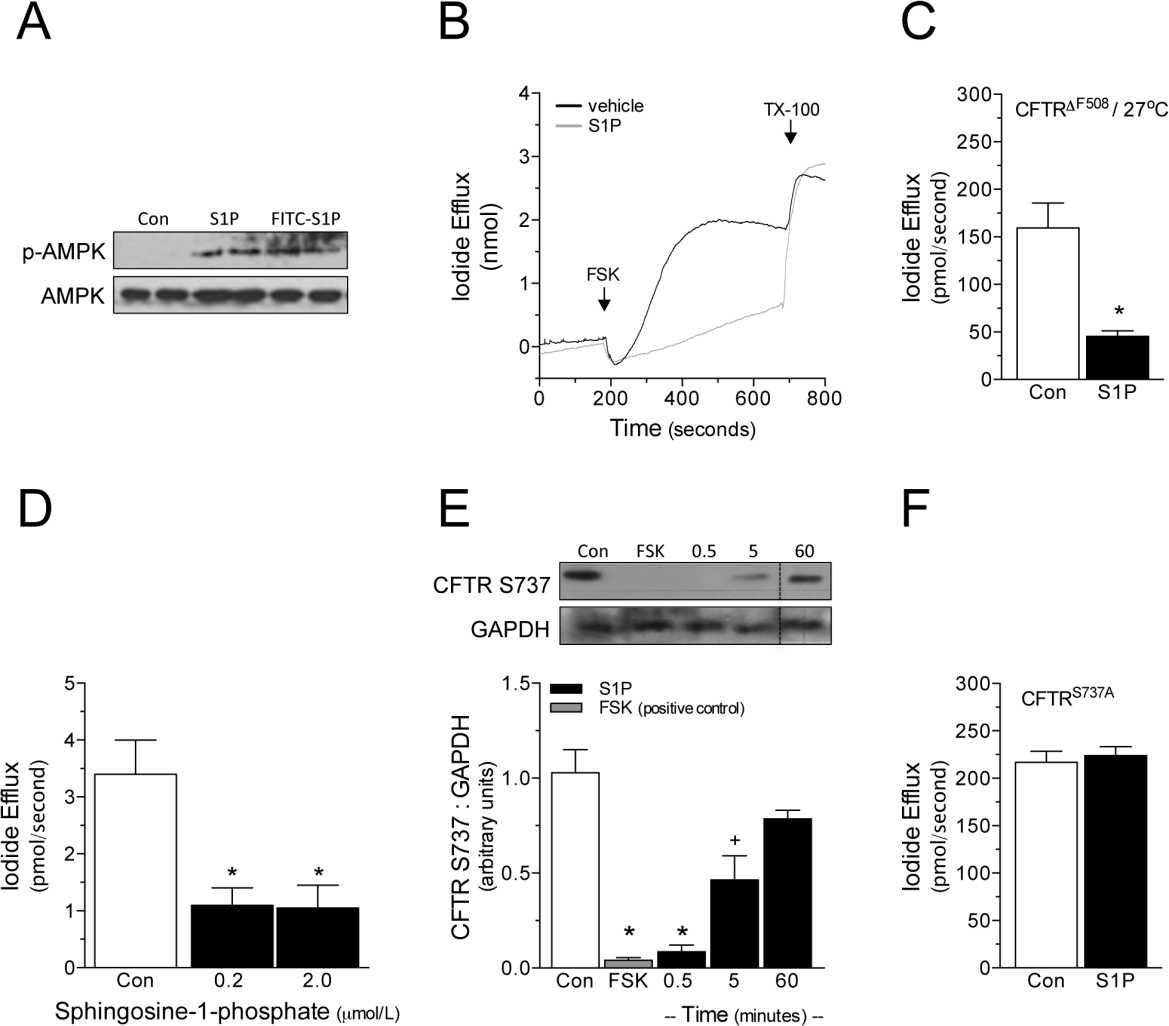
S1P attenuates CFTR-dependent iodide efflux via phosphorylation of serine 737. (A) Shown is a representative western blot demonstrating that 1μmol/L FITC-S1P significantly increases AMPK phosphorylation (control n = 4; FITC-S1P n = 6). The extent of AMPK phosphorylation is qualitatively similar to that elicited by unlabeled S1P (n = 2). (B) Shown is a representative tracing of iodide efflux in BHK cells stably transfected with wild-type CFTR (CFTR^wt^). Forskolin (FSK; 20μmol/L) stimulates robust CFTR-dependent iodide efflux; more iodide is released in the vehicle control (4% BSA), compared to cells treated with 1μmol/L S1P. Permeablization with 0.1% Triton X-100 (TX-100) confirms that iodide loading was similar. (C) S1P (1μmol/L) attenuates iodide efflux in temperature-rescued (27°C for 24h) BHK cells stably expressing the ΔF508 CFTR mutant (CFTR^ΔF508^; n = 6). (D) S1P maximally inhibits iodide efflux at a 200μmol/L concentration in BHK cells stably expressing CFTR^wt^ (n = 7). (E) Western blots utilizing an antibody that exclusively binds non-phosphorylated CFTR serine residue 737 (S737) indicate that CFTR^wt^ is rapidly (within 30 seconds) phosphorylated in response to 1μmol/L S1P (n = 6). The response is transient in nature, with phosphorylation significantly reduced after 5 minutes (n = 6) and fully reversed after 60 minutes (n = 5). FSK (20μmol/L; 5 minutes) also stimulates robust CFTR S737 phosphorylation. (F) S1P fails to inhibit iodide efflux in naïve BHK cells transiently transfected with a plasmid encoding a mutated CFTR containing a serine-to-alanine substitution at amino acid 737 (CFTR^S737A^). * denotes P<0.05 relative to the control; in *Panel E*, + denotes a significant difference between both the control and 0.5 minute S1P treatment groups. *Panels B* and *D* display iodide efflux data collected from cell suspensions (i.e., “real time” measurements); *Panels C* and *F* display iodide efflux data collected from cell monolayers.

As shown in Fig 1A, FITC-S1P (1μmol/L) stimulates robust AMPK phosphorylation in BHK cells expressing CFTR^wt^ (n = 6): this result confirms that FITC-labeled S1P has intrinsic cell-signaling activity, a critical confound that precludes using FITC-S1P uptake to assess S1P-dependent effects on CFTR activity. We therefore measured iodide efflux during maximal CFTR channel activation [19] as the primary means of assessing CFTR activity. Deficient CFTR channel gating (i.e., naïve BHK cells transfected with CFTR^G551D^) [20] abolishes S1P uptake (uptake not statistically different from background, n = 7): since both iodide efflux [20] and S1P uptake require intact CFTR gating, they are likely both sensitive to R-domain regulation.

Iodide efflux is negligible under non-stimulated conditions (0.001±0.001μmol/L, n = 18; not statistically different from zero; also see Panel C of Fig A in S1 File). The CFTR activator forskolin (FSK; 20μmol/L) stimulates significant iodide efflux in BHK cells stably expressing CFTR^wt^ (Fig 1B). To verify the CFTR dependency, we confirmed that (i) FSK does not stimulate iodide efflux in naïve, non-transfected BHK cells, as previously demonstrated [18,21] and (ii) CFTR inhibition (CFTR(inh)-172) effectively abolishes stimulated efflux in CFTR^wt^-expressing BHK cells (by 91±4%, n = 3; P<0.05 relative to vehicle control). CFTR must be expressed at the cell surface in order to release iodide to the extracellular compartment; accordingly, FSK does not stimulate iodide efflux in BHK cells stably expressing CFTR^ΔF508^ (no efflux detected, n = 5) [21], a CFTR mutant that is retained in the endoplasmic reticulum [22] and exhibits a short half-life at the plasma membrane [23]. As previously reported [21–24], low temperature rescue (i.e., culturing cells at 27°C for 24 hours) promotes CFTR^ΔF508^ integration into the plasma membrane and confers FSK the ability to stimulate iodide efflux (n = 6; Fig 1C). Taken together, these experiments establish that iodide efflux in this cell system is a specific measure of maximal cell surface CFTR activity.

S1P (1μmol/L co-stimulation) significantly attenuates FSK-stimulated iodide efflux in CFTR^wt^-expressing BHK cells. Representative tracings are displayed in Fig 1B. The difference in slope shown in Fig 1B establishes that the S1P-stimulated attenuation is functional in nature. Consistent with its effects on wild-type CFTR (Fig 1B), S1P also attenuates FSK-stimulated iodide efflux in temperature-rescued BHK cells expressing CFTR^ΔF508^ (n = 6; Fig 1C). Although we routinely use 1μmol/L S1P as our standard concentration, S1P already maximally inhibits FSK-stimulated iodide efflux at the physiologically relevant [25] concentration of 200nmol/L (n = 6; Fig 1D).

CFTR serine 737 (S737) is phosphorylated by several serine kinases, including PKA [26], AMPK [10] and lemur tyrosine kinase 2 (LMTK2) [27]. In the present study, FSK (a potent PKA activator; 20μmol/L, 30 seconds) served as a positive control for the induction of CFTR S737 phosphorylation: FSK rapidly stimulates maximal CFTR S737 phosphorylation (i.e., abolishes 67D4 antibody binding; Fig 1E and Panel A of Fig B in S1 File), a response that persists for at least 30 minutes (no CFTR detection with 67D4 antibody after 30 minutes FSK treatment, n = 5).

S1P (1μmol/L) stimulates rapid (within 30 seconds) and transient (i.e., statistically less phosphorylation at 5 minutes, relative to 30 seconds) CFTR S737 phosphorylation (n = 5–6; Fig 1E). We confirmed this result using the phospho-sensitive 570 CFTR antibody (Fig C in S1 File) [28]; we additionally validate that normalizing to a standard housekeeping protein provides a reliable measure, thereby eliminating the need for duplicate blots (Fig C in S1 File).

Mutating the CFTR serine 737 phosphorylation site (i.e., serine-to-alanine mutation; CFTR^S737A^) abolishes S1P’s inhibitory effect on FSK-stimulated iodide efflux (Fig 1F), substantiating the crucial role this phosphorylation site plays in the regulatory mechanism. Iodide efflux is negligible in BHK cells expressing CFTR^S737A^ under non-stimulated conditions (i.e., no efflux detected without CFTR channel activation; n = 4).

### S1P attenuates iodide efflux via an S1P receptor / AMPK-dependent mechanism

**Fig 2:**
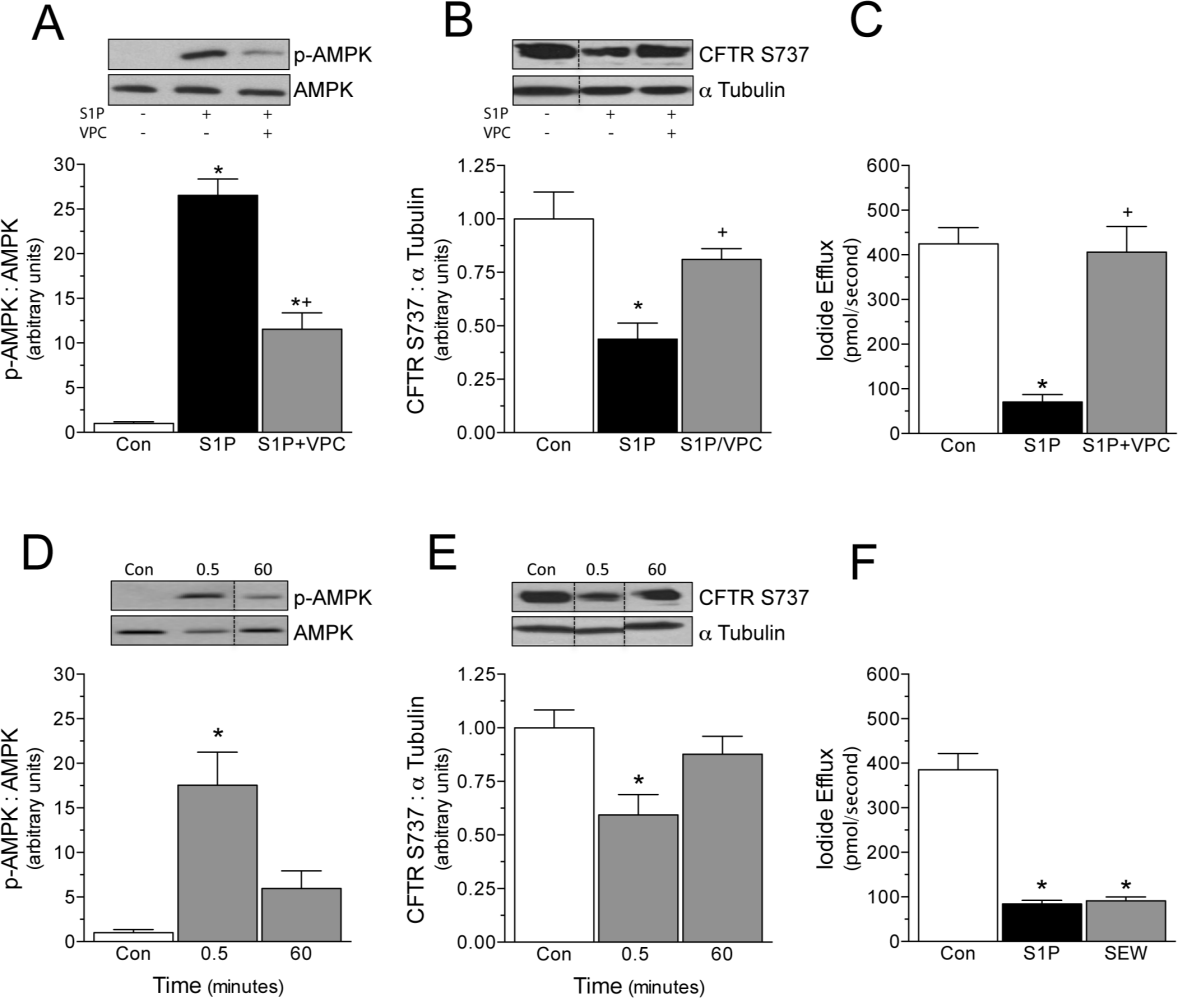
S1P mediates its effects via the S1P_1_ receptor subtype. In BHK cells stably expressing CFTR^wt^, the chemical S1P_**1**_ / S1P_**3**_ receptor antagonist VPC23019 (VPC; 4μmol/L, 30min pre-treatment) attenuates S1P (1μmol/L)-stimulated (A) AMPK phosphorylation (n = 8) and abolishes (B) CFTR S737 phosphorylation (n = 9) and (C) iodide efflux inhibition (n = 6). The S1P_**1**_ receptor-specific agonist SEW-2871 (SEW; 1μmol/L) increases both (D) AMPK phosphorylation (n = 6), (E) CFTR phosphorylation (n = 5). (F) SEW co-stimulation attenuates iodide efflux to a similar extent as S1P (n = 6 for control and SEW; n = 3 for S1P). * denotes P<0.05 relative to the control; In *Panels A-C*, + denotes P<0.05 for VPC+S1P, relative to S1P. All iodide efflux data was collected from cell monolayers.

According to our proposed mechanistic model, extracellular S1P initiates its regulatory effects on CFTR through the activation of a cell surface S1P receptor. Indeed, S1P_1_ / S1P_3_ receptor antagonism (4μmol/L VPC 23019; 30 minutes) attenuates S1P-stimulated AMPK phosphorylation (Fig 2A; n = 3) and abolishes the S1P-dependent effects on CFTR S737 phosphorylation (Fig 2B; n = 9) and iodide efflux (Fig 2C; n = 6) in BHK cells expressing CFTR^wt^. To discern which of two candidate receptors mediates the response, we utilized the S1P_1_ receptor (S1P_1_R)-specific agonist SEW-2871 and the S1P_3_ receptor (S1P_3_R)-specific agonist CYM-5541 (both used at 1μmol/L). Selective S1P_1_R activation (SEW-2871) mimics the effects of S1P: we observe rapid and transient phosphorylation of both AMPK (Fig 2D; n = 6) and CFTR S737 (Fig 2E; n = 5); co-stimulation attenuates FSK-stimulated iodide efflux (Fig 2F; n = 6), to a similar extent as S1P (n = 3). In contrast, selective S1P_3_R activation (CYM-5541) stimulates only mild AMPK phosphorylation (n = 9–10), which does not induce CFTR S737 phosphorylation (Fig D in S1 File).

**Fig 3:**
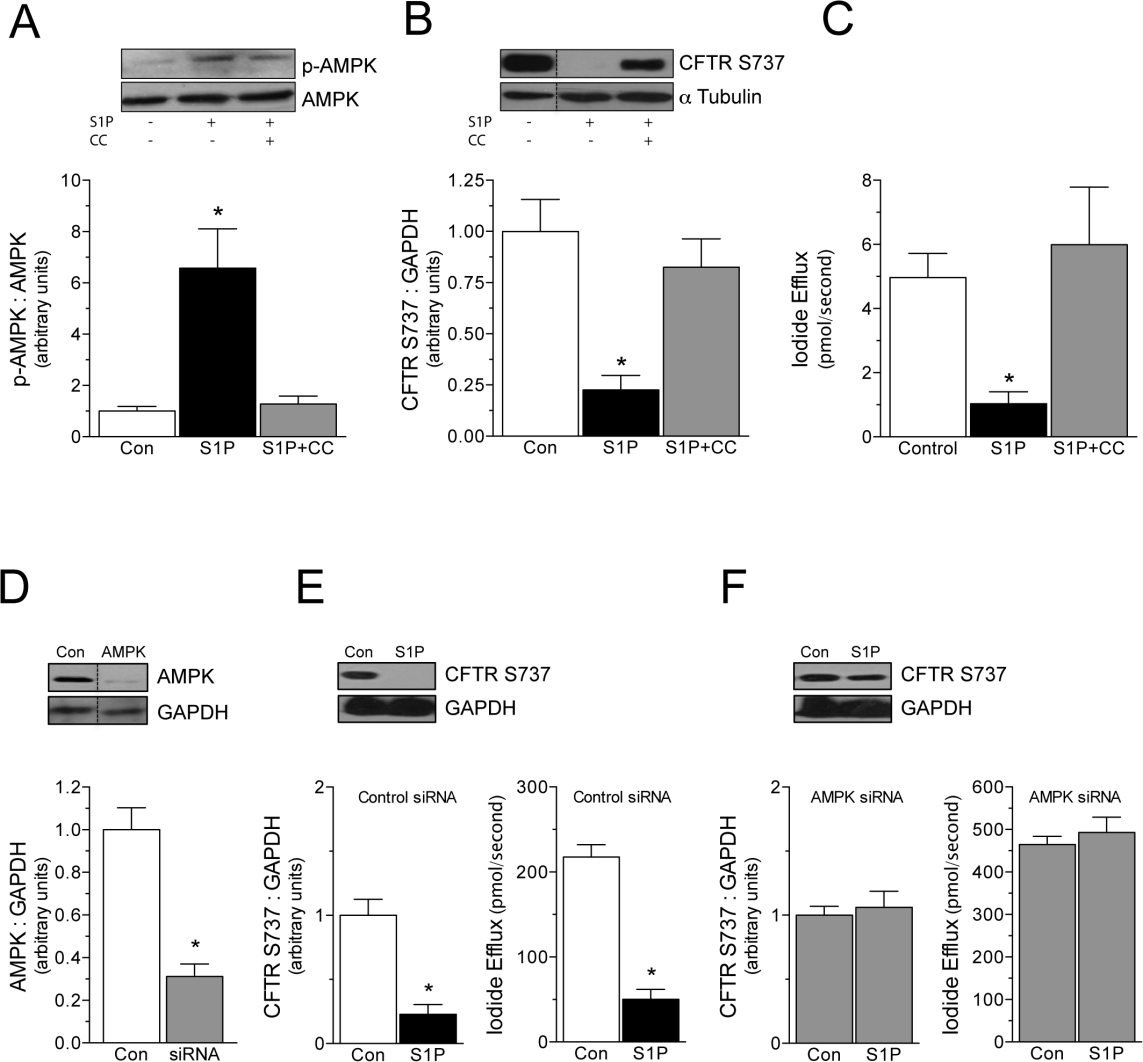
S1P inhibits CFTR by an AMPK-dependent mechanism. In BHK cells stably expressing CFTR^wt^, the chemical AMPK inhibitor compound C (CC; 80μmol/L, 30min pre-treatment) abolishes S1P (1μmol/L)-stimulated (A) AMPK phosphorylation (n = 6), (B) CFTR S737 phosphorylation (n = 6) and (C) attenuates iodide efflux (n = 5–6). (D) Transfecting siRNA targeting the AMPK α1 subunit effectively reduced AMPK α1 protein expression in BHK cells stably expressing CFTR^wt^; control siRNA had no effect on AMPK α1 expression (n = 12). (E) As expected, S1P stimulated CFTR S737 phosphorylation (n = 5–6) and attenuated FSK-stimulated iodide efflux (n = 6) in BHK cells expressing CFTR^wt^. (F) In contrast, S1P had no effect on CFTR S737 phosphorylation or iodide efflux in BHK cells expressing CFTR^wt^ following treatment with AMPK-targeting siRNA (n = 5–6). * denotes P<0.05 relative to the control. *Panel C* displays iodide efflux data collected from cell suspensions (i.e., “real time” measurements); all other iodide efflux data were collected from cell monolayers.

Since CFTR S737 is not exclusively phosphorylated by AMPK [29] (Fig 1E), we next assessed whether AMPK activation is mandatory for S1P-stimulated CFTR phosphorylation and CFTR channel regulation. Using BHK cells expressing CFTR^wt^, we confirmed that the chemical AMPK inhibitor Compound C (80μmol/L; 30 minute pre-treatment) prevents S1P-stimulated AMPK phosphorylation (Fig 3A; n = 6). As expected, AMPK inhibition abolishes S1P-stimulated CFTR S737 phosphorylation (Fig 3B; n = 6) and the attenuation of iodide efflux (Fig 3C; n = 5–7).

We complemented the use of Compound C with a second, more specific inhibitory strategy: siRNA-mediated knockdown of AMPK protein expression. Western blots confirmed that the siRNA targeting human AMPK α1 catalytic subunit mRNA significantly reduce AMPK α1 protein expression in BHK cells expressing CFTR^wt^ (relative to cells receiving a non-targeting control siRNA; Fig 3D; n = 12). In cells treated with non-targeting control siRNA (Fig 3E), S1P stimulated CFTR S737 phosphorylation (n = 5–6) and attenuated FSK-stimulated iodide efflux (n = 6); however, AMPK siRNA abolished both responses (Fig 3F; n = 5–6), consistent with observations for Compound C

### Interrupting S1P / AMPK signaling increases the activity of CFTR^ΔF508^

Our previous work positions CFTR as a negative regulator of S1P signaling [5,6]. Accordingly, pathological reductions in CFTR expression enhance S1P signaling [6], presumably because extracellular S1P levels increase as a result of compromised CFTR-dependent S1P transport. We propose that under these conditions (i.e., reduced CFTR expression), the enhanced S1P signaling activates the S1P/AMPK/CFTR axis to functionally inhibit the remaining CFTR channels. We tested this hypothesis in BHK cells expressing CFTR^ΔF508^, a mutation that profoundly reduces cell surface CFTR abundance. We expect that resulting enhancement of S1P signaling will functionally inhibit the small proportion of CFTR^ΔF508^ channels that localize to the cell surface.

**Fig 4:**
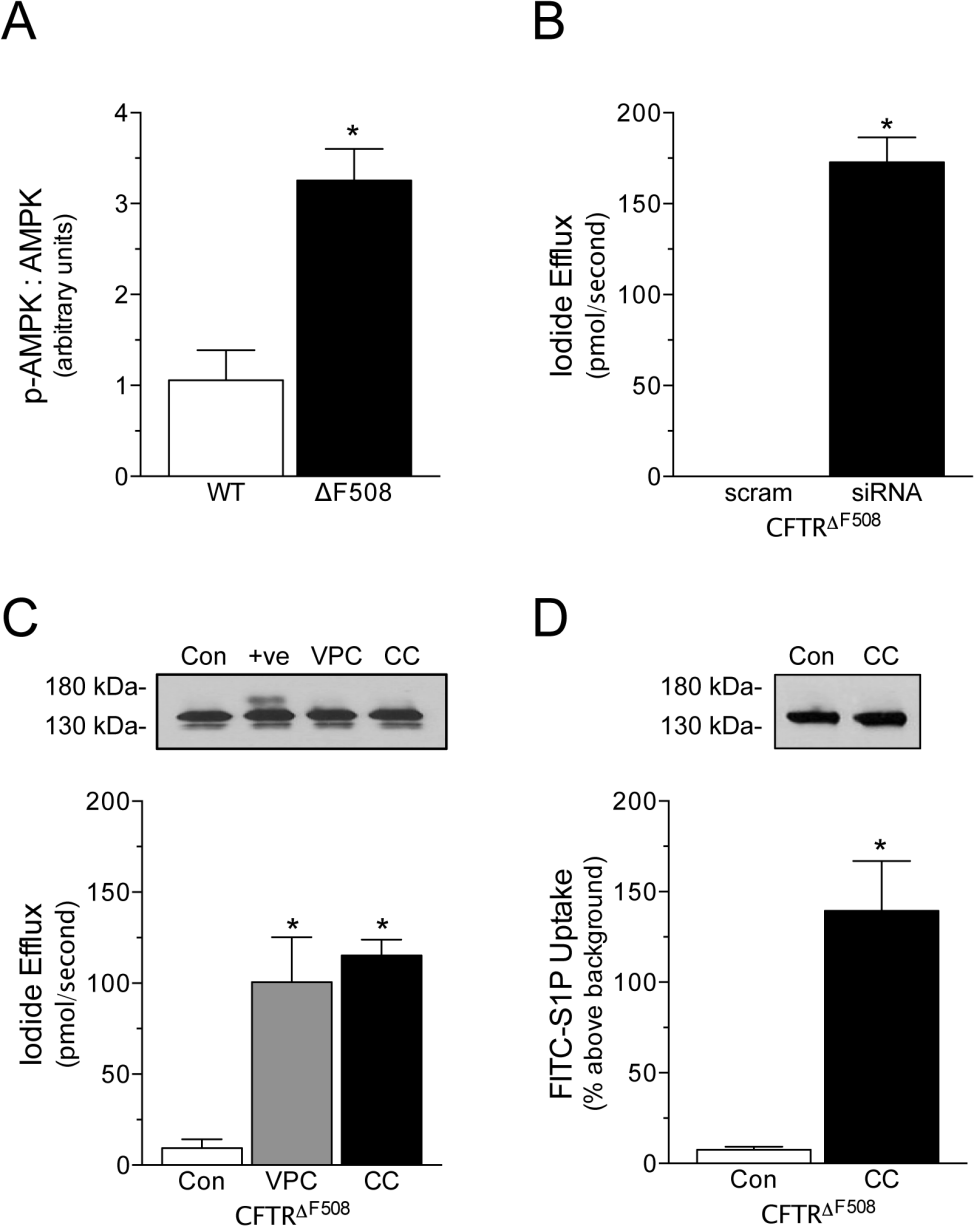
S1P/AMPK signaling functionally inhibits CFTR^ΔF508^ activity. (A) AMPK phosphorylation is higher in mesenteric arteries isolated from mice carrying the ΔF508 CFTR mutation, relative to wild-type littermates (n = 4). (B) Targeted, siRNA-mediated AMPK inhibition (n = 6) augments iodide efflux in BHK cells expressing the ΔF508 CFTR mutant (CFTR^ΔF508^), relative to the scrambled siRNA control (n = 6). (C) Both S1P_**1**_ / S1P_**3**_ receptor antagonism (4μmol/L VPC23019 for 24 hours; VPC; con n = 11, VPC n = 7) and pharmacological AMPK inhibition (1μmol/L Compound C for 24 hours; CC; n = 6) improve iodide efflux in BHK cells expressing CFTR^ΔF508^. Western blots (inset above) show no change in CFTR^ΔF508^ electrophoretic mobility following VPC and CC treatment (n = 3); the corrector VX-809 (3μmol/L for 24 hours; “+ve”; n = 3) acts as a positive control. (D) AMPK inhibition (20μmol/L Compound C) increases FITC-S1P uptake in BHK cells expressing CFTR^ΔF508^ (con n = 10, CC n = 5); western blots (inset above) show no change in CFTR^ΔF508^ electrophoretic mobility following treatment (n = 3). * denotes P<0.05 relative to the control. All iodide efflux data was collected from cell monolayers.

Indeed, AMPK phosphorylation is elevated in mouse mesenteric arteries (i.e., a vascular bed where CFTR deletion augments S1P-dependent responses [6]) isolated from CFTR^ΔF508^ mice, relative to wild type controls (Fig 4A; n = 4). Consistent with our proposed model, specifically reducing AMPK expression (via siRNA) in BHK cells expressing CFTR^ΔF508^ improves iodide efflux (Fig 4B; n = 5–6). In accordance with these results, pharmacologically inhibiting AMPK (1μmol/L Compound C, 24 hours) or antagonizing S1P receptors (4μmol/L VPC 23019; 24 hours) also improves iodide efflux in BHK cells expressing CFTR^ΔF508^ (Fig 4C; n = 6–11). Interestingly, both S1P receptor antagonism and AMPK inhibition (Fig 4C) improve iodide efflux to a similar extent as low-temperature rescue (P = N.S. comparing temperature rescue in Fig 1C, VPC 23019 in Fig 4C, Compound C in Fig 4C and AMPK siRNA in Fig 4B by Kruskal-Wallis non-parametric one-way ANOVA). Western blots indicate negligible CFTR^ΔF508^ correction following pharmacological treatment (Fig 4C, n = 3), consistent with a functional, rather than trafficking effect. VX-809 (a CFTR corrector compound; 3μmol/L for 24 hours) served as a positive control for correction (Fig 4C; n = 3).

Improving CFTR^ΔF508^ activity should result in the transport of labeled S1P across the plasma membrane (i.e., as shown for low-temperature rescue [6]). Since FITC-S1P uptake assessments could be confounded by VPC 23019 (an S1P analogue), we assessed whether Compound C (20μmol/L, 5 minute pre-treatment), which has no obvious confound, improves S1P transport in BHK cells expressing CFTR^ΔF508^: as expected, AMPK inhibition significantly enhances FITC-S1P uptake (Fig 4D; n = 5–10). Consistent with the results in Fig 4C, western blots indicate negligible CFTR^ΔF508^ correction following treatment (Fig 4D; n = 3).

## Discussion

The present investigation demonstrates that S1P regulates CFTR activity (defined here as the product of surface expression and channel gating). Given the ubiquitous and highly integrative nature of S1P signaling [14], this seminal discovery potentially links a multitude of S1P effector pathways to CFTR channel function. This potential feedback link between S1P and CFTR builds a “regulatory tandem” that amplifies S1P signals; under pathological settings, this mechanism may exaggerate a reduction in CFTR activity.

We strategically selected the BHK expression system to address our mechanistic hypothesis, so that human CFTR constructs (a necessity, since the phospho-sensitive CFTR antibody targets a human epitope) could be expressed in an environment with minimal endogenous CFTR expression. Although BHK cells do not natively express CFTR, the S1P and AMPK signaling pathways are ubiquitous and have highly conserved links in virtually all cell types [11,14]. Indeed, the S1P_1_R-dependent AMPK activation we document in the present study matches observations from a distinctly separate model of cultured endothelial cells [8]. Further, the rapid and transient CFTR phosphorylation kinetic described in the present study is similar to the one described for S1P-stimulated AMPK phosphorylation in endothelial cells [7]. Taken together, the agreement between the two cell systems confers confidence that results derived from BHK cells are broadly applicable. Nevertheless, the artificial nature of heterologous expression systems requires due caution with respect to transferring the physiological implications to other cell types and obliges further investigation in models that more closely mimic *in vivo* settings.

Iodide efflux provides a rapid, convenient and sensitive assessment of cell surface CFTR channel activity [18,30,31]. Although the continuous and discontinuous measurement approaches produce strikingly different absolute efflux measures (e.g., compare Fig 1D and Fig 2C), the discrepancy is eliminated by normalization to total iodide loading (at “plateau” following control stimulation, both methods flux 70% of the total iodide loaded). This indicates a technical reason, rather than an alteration in cellular signaling, underlies the difference in iodide efflux magnitude. To our knowledge, iodide efflux measures have not been used to assess the modulatory effects of S1P or AMPK on CFTR channel activity; however, our data align well with previous electrophysiological data characterizing AMPK as a modulator of CFTR channel gating [10,32].

S1P markedly attenuates PKA-stimulated CFTR channel activity, with requisite involvement of S1P_1_R, AMPK and CFTR S737 phosphorylation. Although antagonizing S1P_1_R/S1P_3_R (VPC23019) prevents exogenously-applied S1P from attenuating CFTR activity, receptor antagonism alone (i.e., in the absence of exogenously applied S1P) has no effect: BHK cells expressing CFTR^wt^, therefore, must possess sufficient S1P transport capacity to sequester all constitutively produced/released S1P (i.e., S1PR activation is minimal under basal conditions). S1P exerts maximal effects on CFTR channel activity at the physiologically relevant concentration (200nmol/L) [25].

CFTR S737 phosphorylation is crucial for attenuating CFTR activity and yet, this does not entirely explain the inhibitory effect: as shown in Fig 1E, FSK also rapidly phosphorylates CFTR S737. Since FSK is used to stimulate iodide efflux, CFTR S737 is undoubtedly phosphorylated in the control setting (i.e., in the absence of S1P stimulation). Thus, to inhibit CFTR, AMPK must ***both*** phosphorylate and persistently associate with CFTR: if the AMPK α1 subunit is mutated to reduce either kinase activity (i.e., binds to CFTR, but does not phosphorylate) or CFTR interaction (i.e., phosphorylates CFTR, but does not maintain persistent binding), then AMPK is rendered incapable of attenuating CFTR channel activity [32]. Thus, the S1P-stimulated CFTR inhibition documented in the present investigation putatively results from the combined allosteric effects of CFTR R-domain phosphorylation and CFTR-AMPK protein interaction.

To function properly, the system must maintain a delicate balance: with high CFTR activity, even stimulated S1P signals would be silenced; conversely, if CFTR activity is too low or compromised (e.g., through mutation [22] or pathologically reduced CFTR expression [6]), the unmasking of endogenous S1P signals could initiate a vicious feedback cycle that then persistently attenuates CFTR activity. We tested this hypothesis using BHK cells expressing CFTR^ΔF508^, where only a small proportion of CFTR^ΔF508^ localizes to the cell surface [33,34]. As predicted, the enhanced S1P signaling (putatively resulting from less degradation) associated with the ΔF508 CFTR mutation elevates AMPK activity, which functionally antagonizes the proportion of CFTR^ΔF508^ residing at the cell surface (Fig 4).

The high level of CFTR^ΔF508^ activity observed following S1P/AMPK signaling inhibition is surprising, given the absence of classical correction mechanism (i.e., no measurable increase in Band C, the fully mature CFTR form that is widely accepted to mediate iodide efflux). Several factors may contribute to this observation: as examples, it is possible that (i) the small fraction of CFTR^ΔF508^ channels localized to the cell surface [17] is more highly activated than CFTR^wt^ when S1P/AMPK signaling is inhibited; and/or (ii) non-canonical transport mechanisms localize the Band B form (i.e., core-glycosylated CFTR^ΔF508^) to the plasma membrane [35]. Interestingly, the latter would imply that Band C is not the sole conducting form of CFTR when S1P/AMPK signaling is inhibited.

The discovery that S1P/AMPK signaling shuts down the CFTR^ΔF508^ channels that reside at the cell surface has significant clinical ramifications, given that most the prevalent form of cystic fibrosis is caused by the ΔF508 mutation. Therapeutics currently in development primarily target the defect of insufficient trafficking to the membrane. However, the ultimate efficacy of these correction compounds is limited, with rescued CFTR^ΔF508^ expression/activity generally representing only 10–30% of the wild-type level [36–38]. S1P_1_ receptor modulators are clinically available (e.g., fingolimod) and second-generation compounds are currently in clinical trials [39]. Exploiting these modulators’ ability to interfere with the S1P/AMPK signaling link and thereby improve CFTR^ΔF508^ channel activity (Fig 1C, Fig 4) could synergistically enhance the efficacy of correction therapeutics. In fact, our data show that S1PR and AMPK inhibition significantly restore CFTR activity in CFTR^ΔF508^ expressing cells, even in the absence of correction.

Our previous work demonstrates that heart failure induces widespread CFTR expression reductions (e.g., in posterior cerebral arteries, lung epithelium and heart tissue); we therefore proposed that CFTR therapeutics possess unexplored utility for treating heart failure-associated vascular dysfunction and multi-organ injury [6]. Analogous to the consequences of deficient CFTR trafficking (i.e., CFTR^ΔF508^), inflammatory-based CFTR down-regulation should reduce S1P degradation, thereby activating the S1P/AMPK signaling pathway and ultimately inhibit the residual CFTR channels. Indeed, elevated S1P levels (Fig E in S1 File) and enhanced S1P signaling [6,40] are evident in heart failure. Thus, in addition to possessing therapeutic potential for treating cystic fibrosis, antagonizing S1P/AMPK signaling may possess clinical utility for inflammatory pathologies that down-regulate CFTR [6].

In summary, we demonstrate the presence of a S1P/AMPK signaling mechanism that transiently attenuates CFTR activity and positively reinforces the S1P signal through the suspension of S1P degradation. This new signaling connection could enable a myriad of upstream cellular signals to converge on CFTR, through these ubiquitous and highly conserved signaling foci. Pathological reductions in CFTR expression/activity trigger a vicious cycle of the mechanism’s inhibitory actions: our data, therefore, support complementing CFTR correction/potentiation strategies with the inhibition of S1P/AMPK signaling for greater efficacy. Our mechanistic concept offers the intriguing premise that pharmacological S1P_1_R blockade exceeds simple antagonism at the receptor level: it also unleashes S1P degradation via CFTR disinhibition.

## Supporting Information

S1 File Supplemental Methods and Results. This complete supplemental information file contains: (i) information about reagents; (ii) methodological details, including custom CFTR antibody production and validation, western blot procedures, heart failure induction and S1P quantification; and (iii) supplemental data figures A-E. Supplemental data figures include: Measuring CFTR-dependent iodide efflux (Fig A); Validation of the 67D4 antibody for serine 737 phosphosensitivity (Fig B); Antibody 570 detects S1P-dependent CFTR phosphorylation (Fig C); S1P_3_ receptor activation does not induce CFTR S737 phosphorylation (Fig D); and Lung S1P levels in mice with heart failure (Fig E).
(PDF)
