# Supplementary material for: Sphingosine-1-Phosphate Is a Novel Regulator of Cystic Fibrosis Transmembrane Conductance Regulator (CFTR) Activity
Source: PLoS One. 2015 Jun 16;10(6):e0130313. doi: 10.1371/journal.pone.0130313 (PMC4469317; doi:10.1371/journal.pone.0130313)
Supplement: S1 File — This complete supplemental information file contains: (i) information about reagents; (ii) methodological details, including custom CFTR antibody production and validation, western blot procedures, heart failure induction and S1P quantification; and (iii) supplemental data figures A-E. Supplemental data figures include: Measuring CFTR-dependent iodide efflux (Fig A); Validation of the 67D4 antibody for serine 737 phosphosensitivity (Fig B); Antibody 570 detects S1P-dependent CFTR phosphorylation (Fig C); S1P3 receptor activation does not induce CFTR S737 phosphorylation (Fig D); and Lung S1P levels in mice with heart failure (Fig E). (PDF) [file pone.0130313.s001.pdf]

Supplemental Methods and Results

Reagents

Sphingosine-1-phosphate (S1P) was purchased from Enzo Life Sciences, Inc. (distributed by Cedarlane Laboratories, Burlington Canada); fluorescein-labeled S1P (FITC-S1P) from Echelon Biosciences (Cedarlane laboratories); VX-809 (Lumacaftor) from Selleck Chemicals (Cedarlane Laboratories) and the $S1P_1/S1P_3$ receptor antagonist VPC-23019, the selective $S1P_1$ receptor agonist SEW-2871, the selective $S1P_3$ receptor agonist CYM-5541 and the AMPK inhibitor Compound C (dorsomorphin dihydrochloride) were purchased from Tocris Bioscience (Cedarlane Laboratories). Unless otherwise specified, all other chemical reagents were purchased from Sigma-Aldrich.

Dr. John Riordan (University of North Carolina - Chapel Hill) provided two CFTR antibodies through the *Cystic Fibrosis Foundation Therapeutics* Antibody Distribution Program (http://www.cff.org/research). Antibody "570" targets CFTR amino acids 731-742 (i.e., includes the S737 phosphorylation site) [1] and is phosphorylation sensitive [2]; antibody "596" targets amino acids 1204-1211 (i.e., located in nucleotide binding domain 2; NBD2) [1]. Dr. David Thomas (McGill University, Montreal, Canada) generously provided 2 custom-made monoclonal antibodies that target CFTR's regulatory domain (designated "67D4" and "22E8"). All other antibodies utilized in this study are commercially available.

Custom CFTR Antibody Production

The "67D4" and "22E8" custom-made monoclonal antibodies (Dr. David Thomas; McGill University, Montreal, Canada) were prepared using a previously described technique for monoclonal antibody generation [3]. Using a standard immunization protocol, mice (strain: SJL) were immunized with 75µg of bacterially expressed/purified "6His-R-domain CFTR" (a peptide encompassing human CFTR's Nucleotide Binding Domain 1, Regulatory Domain and Nucleotide Binding Domain 2) that was emulsified in Titermax gold adjuvant (Cedarlane Laboratories). After the third immunization, spleen cells were fused with Sp-2/0 myeloma cells. Hybridoma lines were identified by ELISA using *E. coli* expressed 6His-R-domain CFTR as an antigen (50ng/well). Following culture, monoclonal antibodies were purified using CBinD L-coated beads (Sigma) and mapped against a peptide array (biotinylated 15-mer peptides; 10 amino acid overlap between peptides; bound to streptavadin-coated plates).

The antibody derived from clone 67D4 recognizes the epitope ^{726}EDSDEPLEPPL**S**LVP^{740}, which includes the serine 737 phosphorylation site; the antibody derived from clone 22E8 recognizes the epitope ^{756}STGPTLQARR^{765}, a sequence that is not predicted to contain a phosphorylation site.

Validation of the 67D4 phospho-sensitive CFTR Antibody

In Baby Hamster Kidney (BHK) cells stably expressing $CFTR^{wt}$, forskolin (FSK; 20µmol/L, 30 seconds) abrogates 67D4 CFTR antibody binding; detection with the M3A7 CFTR antibody, which targets CFTR's nucleotide binding domain 2 (NBD2), is not affected (Panel A of Fig B). To conclusively confirm the 67D4 CFTR antibody's specificity and phosphorylation sensitivity, we utilized a freshly purified CFTR R-domain peptide (corresponding to CFTR amino acids 654-838; generously provided by Dr. Zoltan Bozoky and Dr. Julie Forman-Kay; Hospital for Sick Children, Toronto, Canada) [4]. The peptide was provided in an unphosphorylated state: a proportion was phosphorylated *in vitro* with Protein Kinase A (PKA; 0.2 mg/mL R-domain peptide incubated for 5 hours at 37°C in buffer 50mmol/L Tris-HCl (pH 7.5), 150mmol/L NaCl, 2mmol/L dithiothreitol, 200nmol/L PKA). As expected, the 67D4 CFTR antibody detects the non-phosphorylated R-domain peptide, but not the PKA-phosphorylated form (Panel B of Fig B). In contrast, the 22E8 CFTR antibody, which targets an R-domain epitope not predicted to contain a phosphorylation site, detects both the non-phosphorylated and PKA-phosphorylated peptide (Panel B of Fig B).

Since BHK cells stably expressing $CFTR^{wt}$ predominantly display fully-glycosylated CFTR (Band C), less mature forms (i.e., Bands A and B) are more difficult to detect. HEK cells transiently transfected with $CFTR^{wt}$, however, clearly display CFTR Bands A, B and C (Panel C of Fig B). The 67D4 CFTR antibody detects all CFTR glycosylation states (i.e., Bands A, B and C), as does the 596 CFTR antibody that targets CFTR NBD2 (Panel C of Fig B). Comparing blots from HEK cells transiently expressing $CFTR^{\Delta F508}$ and $CFTR^{wt}$ confirms that the Band C detected in lysates from cells expressing $CFTR^{wt}$ is specific to CFTR (Panel C of Fig B).

Using a standard site-directed mutagenesis technique, we mutated serine 737 in our $CFTR^{\Delta F508}$ construct to an aspartic acid, using 5′-GAGAGAAGGCTGGACTTAGTACCAGATTC (sense) and 5′-GAATCTGGTACTAAGTCCAGCCTTCTCTC-3′ (antisense) as mutagenic primers; sequencing confirmed the successful mutation. The mutation abolishes 67D4 CFTR antibody binding, but the protein remains immunogenic for an antibody targeting CFTR's NBD2 ("596"; Panel C of Fig B).

Taken together, these data confirm that the custom-made 67D4 CFTR antibody recognizes a non-phosphorylated CFTR epitope containing the serine 737 phosphorylation site.

Western Blotting

Standard western blotting procedures were followed. Briefly, cell were lysed in lysis buffer containing 50 mM Tris-HCl (pH 7.4), 150 mM NaCl, and 1 mM EDTA, 0.1% SDS, 0.1% Triton X-100 and protease inhibitors (Complete-Mini, EDTA-free; Roche Applied Science, Laval, Canada) for 15 minutes on ice. The lysates were centrifuged and the supernatant was combined (4:1 v/v) with 5x Lamelli's sample buffer. Protein samples were electrophoretically resolved on SDS-polyacrylamide gels and transferred to polyvinyldifluoride (PVDF) membranes. The membranes were blocked for 30 minutes with 5% blotting grade skim milk in phosphate-buffered saline (PBS) containing 0.1% Tween-20 (PBS-T).

With the exception of GAPDH, all antibody incubations were carried out overnight at 4^oC. The antibodies and dilutions utilized under these conditions include: mouse monoclonal anti-CFTR (clone #M3A7; targets NBD2; 1:2,000 dilution in 5% milk/PBS-T; Millipore Canada; Etobicoke, Canada); "67D4" mouse monoclonal anti-CFTR (targets R-Domain; 1:1,000 dilution in 5% milk/PBS-T); "22E8" mouse monoclonal anti-CFTR (targets R-Domain; 1:1,000 dilution in 5% milk/PBS-T); "596" mouse monoclonal anti-CFTR (targets NBD2; 1:20,000 dilution in 5% milk/PBS-T); "570" mouse monoclonal anti-CFTR (R-Domain; 1:15,000 dilution in 5% milk/PBS-T); rabbit polyclonal anti-AMPKα (1:2,000 dilution in 2% bovine serum albumin [BSA]/PBS-T; Cell Signaling Technology [CST] cat#2532; Cedarlane Laboratories); rabbit monoclonal phospho(Thr172)-AMPKα (clone D79.5E; 1:1,000 dilution in 2% BSA/PBS-T; CST via Cedarlane Laboratories), mouse monoclonal anti-αtubulin (clone #DM1A; 1:5,000 dilution in 5% milk/PBS-T; CST via Cedarlane Laboratories). Following the primary antibody incubation, the blots were washed with PBS-T and conjugated with the appropriate peroxidase-labeled secondary antibody (1:10,000 in 5% skim milk/PBS-T; GE Healthcare Life Sciences cat# NA931 or NA934; Baie d'Urfe, Canada) for 1-2 hours at room temperature. Blots for GAPDH used a peroxidase-labeled monoclonal primary antibody (clone GAPDH 71.1; 1:15,000 dilution in 5% milk/PBS-T; 2 hour incubation at room temperature; Sigma) and did not require conjugation with a secondary antibody. Standard chemiluminescence procedures were used to expose X-ray film; developed films were evaluated densitometrically using "Image J" software.

Human Embryonic Kidney Cell Culture

Human Embryonic Kidney 293-T (HEK) cells (from American Type Culture Collection [ATCC]; Manassas, USA) were used only for the validation of the 67D4 CFTR antibody. They were maintained in DMEM media containing 10% fetal bovine serum under standard culture conditions (37°C, 5%CO_2). Cells were transiently transfected with CFTR plasmid constructs using PolyFect transfection reagent (Qiagen Canada; Toronto, Canada), according the manufacturer's instructions.

Heart Failure Induction

This investigation conforms to the *Guide for the Care and Use of Laboratory Animals* published by the NIH (Publication No. 85-23, revised 1996). All animal care and experimental protocols were approved by the Institutional Animal Care and Use Committee at the University of Toronto and were conducted in accordance with Canadian animal protection laws. Commercially available wild-type mice (2-3 months; C57BL/6N) were purchased from Charles River Laboratories (Montreal, Canada); they were housed under a standard 14h:10h light-dark cycle, fed normal chow and had access to water *ad libitum*.

Heart failure was induced by surgical ligation of the left anterior descending (LAD) coronary artery [5]. Briefly, mice were anaesthetized with isoflurane, intubated with a 20-gauge angiocatheter and ventilated with room air. Under sterile conditions, the thorax and pericardium were opened, and the LAD was permanently ligated with 7-0 silk suture (Deknatel; Fall River, USA). In sham-operated controls, the thorax and pericardium were opened, but the LAD was not ligated. Following the procedure, the chest was closed and the mice were extubated upon spontaneous respiration. Mice received buprenorphine (an opioid analgesic; 0.1mg/kg twice daily s.c.) for 2 days post-surgery. At 6 weeks post-LAD ligation, tissue samples were collected. Mice were fully anesthetized with isoflurane and humanely euthanized by decapitation immediately prior to collection.

Tissue Sphingosine-1-Phosphate Quantification

Lung tissue S1P levels were assessed by liquid chromatography electrospray ionization tandem mass spectrometry (LC/ESI/MS/MS), as previously described [6]. Briefly, lung tissue was homogenized in water (100mg/ml); using a Biomek FX workstation (Beckman Coulter; Brea, USA), lipids were extracted with organic solvents, lyophilized and reconstituted in methanol/water/formic acid (80:20:0.4, v/v).

The reconstituted lipids were analyzed using a TSQ Quantum Ultra-triple quadrupole mass spectrometer (Thermo Fisher; San Jose, USA), equipped with an electrospray ionization (ESI) probe and interfaced with an Agilent 1100 HPLC (Agilent Technologies; Wilmington, USA). Lipid extracts were separated with a Xbridge C8 column (2.1x30 mm, 3.5 µm; Waters; Milford, USA). Mobile phase A was: MeOH/H_2O/$CHCl_3$/Formic acid (55:40:5:0.4% by v/v), and mobile phase B was: MeOH/Acetonitrile/$CHCl_3$/Formic acid (48:48:4:0.4% by v/v).

Mass spectrometric analyses were performed online using electrospray ionization tandem mass spectrometry in the positive multiple reaction monitoring (MRM) mode. Samples were extracted using a one-phase extraction method (Methanol-Dichloromethane) with internal standards. S1P was quantified by the ratio of analyte to internal standard and a calibration curve obtained by serial dilution of S1P.

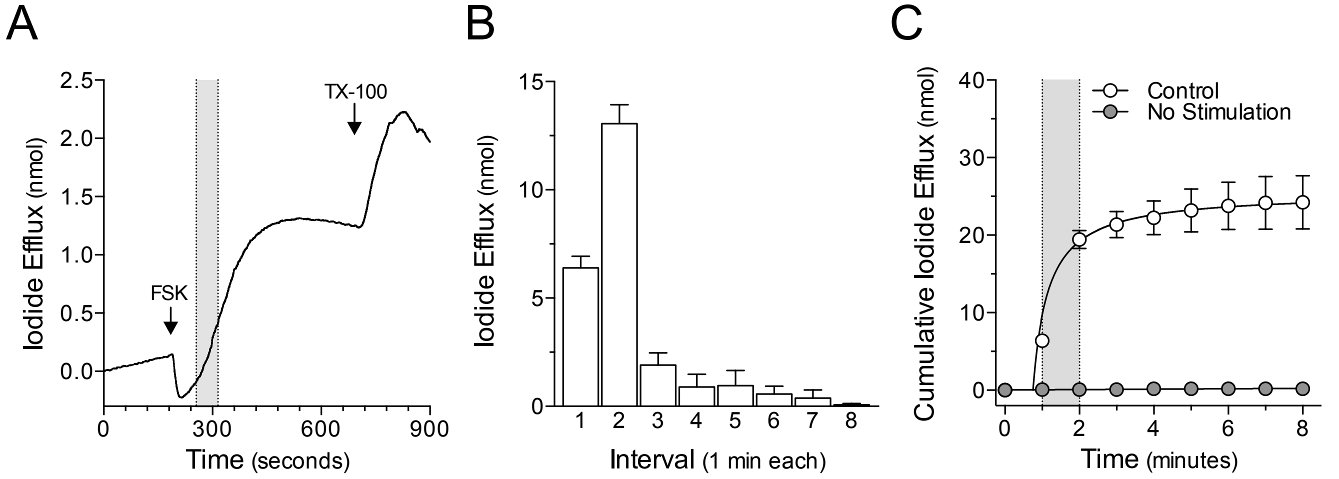

Fig A. Measuring CFTR-dependent iodide efflux.

(A) Shown is a representative "real-time" tracing of extracellular iodide levels measured in a BHK cell suspension (BHK cells express $CFTR^{wt}$). An increasing iodide level (efflux) is evident within 30 seconds of forskolin stimulation (FSK; 20μmol/L); the efflux rate remains constant for at least 2 minutes (linear regression from 1-3 minutes post-FSK yields an r^2 value of 0.997) and then plateaus. The cell suspension is permeablized by the addition of 0.1% Triton X-100 (TX-100), which determines the total loaded iodide in the system (and indirect measure of the number of intact cells). Real-time efflux rates are reported as the slope between 1-2 minutes post-FSK (grey column). (B) Using a modified approach, supernatant iodide levels (cell free) are measured after confluent monolayers of BHK cells expressing $CFTR^{wt}$ (n=6) are sequentially transferred through 8 separate volumes stimulatory efflux buffer for 1 minute each (the buffer contains 10μmol/L FSK, 1mmol/L isobutylmethylxanthine [IBMX] and 100μmol/L cpt-cAMP). (C) The data in *Panel B* are plotted as cumulative iodide efflux, which displays the kinetic more appropriately: stimulated efflux occurs within 1 minute of stimulation and remains high for 1 minute, after which plateauing occurs. Efflux rates in these experiments, therefore, are calculated from the iodide concentration measured in interval 2 of *Panel B* (highlighted in *Panel C* with a grey column). In the absence of stimulation (efflux buffer without FSK, IBMX and cpt-cAMP), BHK cells expressing $CFTR^{wt}$ do not release detectable amounts of iodide.

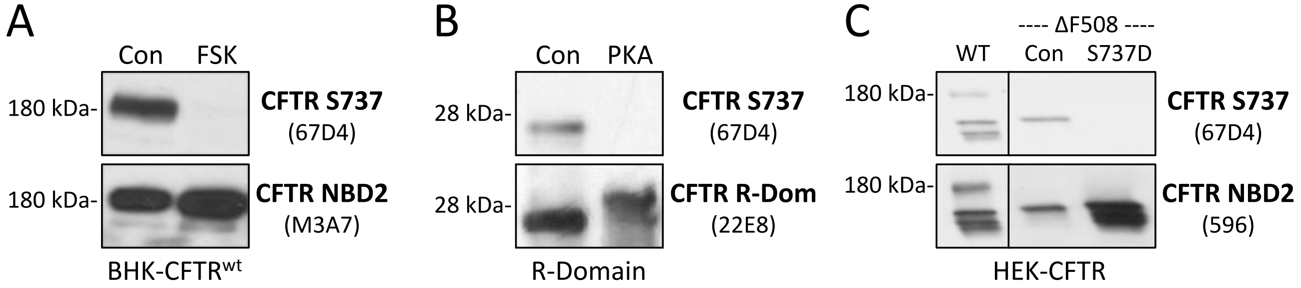

Fig B. Validation of the 67D4 antibody for serine 737 phosphosensitivity.

(A) Shown are representative duplicate western blots of BHK cell lysates (stably expressing wild type CFTR) probed with either the 67D4 (targets R-Domain epitope containing S737) or M3A7 (targets nucleotide binding domain 2; NBD2) CFTR antibody (n=4). Under control conditions, both antibodies robustly detect CFTR (predominantly the fully glycosylated Band C form). Forskolin treatment (FSK; 20μmol/L, 30 seconds) abolishes 67D4 antibody binding, but not M3A7 antibody binding. (B) Shown are representative duplicate western blots of purified CFTR R-domain peptide probed with either the 67D4 or 22E8 (targets R-domain epitope without a phosphorylation site) CFTR antibody (n=2). Both antibodies detect the non-phosphorylated R-domain peptide; protein kinase A (PKA) treatment abolishes 67D4 antibody binding, but not 22E8 antibody binding. Notably, PKA treatment induces an electrophoretic mobility shift of the peptide (discerned with the 22E8 antibody). (C) Shown are representative duplicate western blots of HEK cell lysates (transiently transfected with CFTR expression constructs) probed with either the 67D4 or 596 (targets NBD2) CFTR antibody (n=3). Both antibodies display similar CFTR glycosylation patterns from cell lysates that express wild-type CFTR (WT; Bands B and C detected) or the ΔF508 CFTR mutant (ΔF508/Con; only Band B detected). Mutating the ΔF508 CFTR construct at serine 737 (serine to aspartic acid; ΔF508/S737D) abolishes 67D4 antibody binding, but not 596 antibody binding.

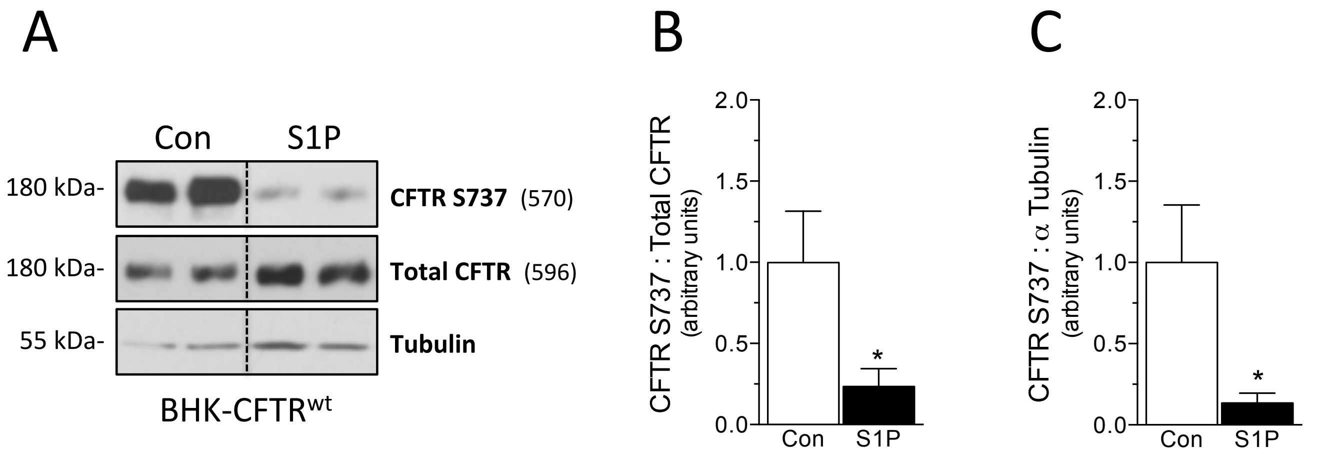

Fig C. Antibody 570 detects S1P-dependent CFTR phosphorylation.

(A) Shown are representative western blots of BHK cell lysates (stably expressing wild type CFTR) probed with the 570 CFTR antibody (targets R-Domain epitope containing S737; CFTR S737), the 596 CFTR antibody (targets NBD2; Total CFTR) and an α-tubulin antibody (a protein typically used for loading normalization). Sphingosine-1-phosphate (S1P; 1μmol/L, 30 seconds) attenuates 570 CFTR antibody binding, but not 596 CFTR antibody binding. Desitometric analyses indicate similar results when the 570 CFTR antibody binding is normalized to (B) Total CFTR (596 antibody binding) or (C) tubulin. * denotes a significant difference; n=5 for both groups.

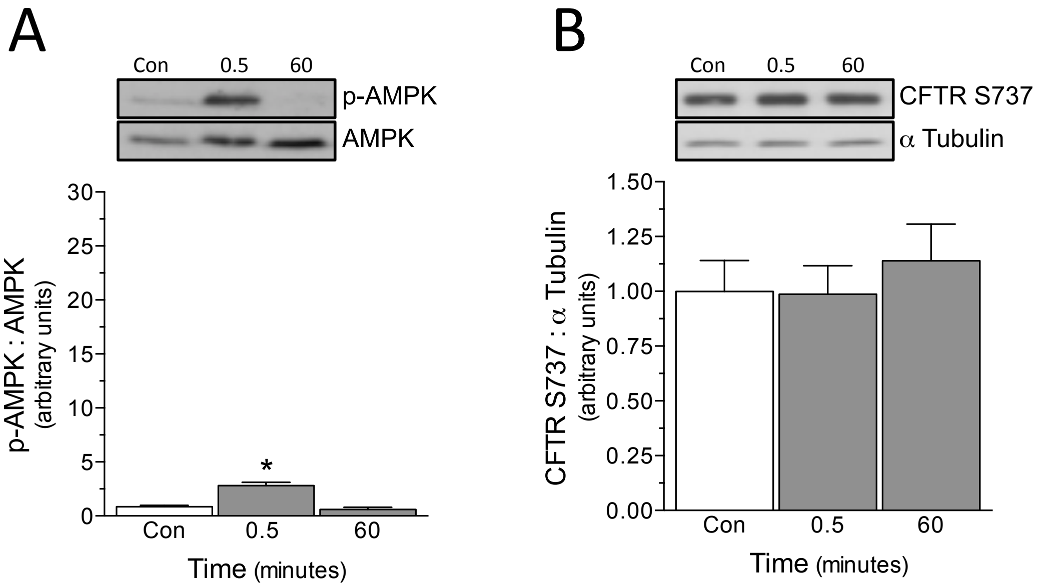

Fig D. S1P$_3$ receptor activation does not induce CFTR S737 phosphorylation.

(A) In BHK cells stably expressing CFTR^{wt}, the S1P$_3$ receptor-specific agonist CYM-5541 (1µmol/L) rapidly (within 30 seconds) stimulates a clear, but small increase in AMPK phosphorylation (n=9-10). AMPK phosphorylation returns to the control level after 60 minutes of stimulation. (B) CYM-5541 does not stimulate CFTR S737 phosphorylation (n=12). * denotes $P<0.05$ for multiple unpaired comparisons to the control.

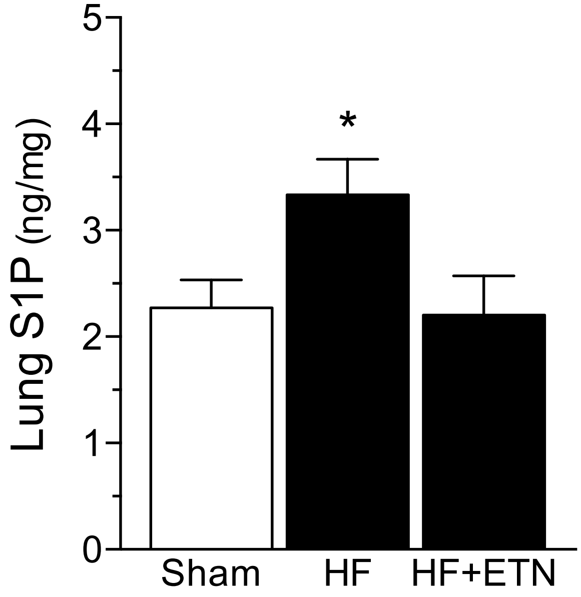

Fig E: Lung S1P levels in mice with heart failure.

In wild-type mice, lung sphingosine-1-phosphate (S1P) levels are significantly elevated in mice with heart failure (HF; 8 weeks post-infarction; n=11), relative to sham-operated controls (n=7). Tumor necrosis factor alpha (TNFα) sequestration via systemic etanercept (ETN) treatment (1 mg/ml s.c. twice per week; treatment initiated immediately following the left anterior descending artery ligation procedure) abolishes the S1P elevation (n=9). * denotes a significant difference relative to the sham-operated control.

Supplement References

1 Cui L, Aleksandrov L, Chang XB, Hou YX, He L, Hegedus T, et al. Domain interdependence in the biosynthetic assembly of CFTR. J Mol Biol. 2007;365: 981-994.

2 Pasyk S, Molinski S, Ahmadi S, Ramjeesingh M, Huan LJ, Chin S, et al. The major cystic fibrosis causing mutation exhibits defective propensity for phosphorylation. Proteomics. 2015;15: 447-461.

3 Veliceasa D, Tauscher G, Surányi G, Kós PB, Likó I, Santore U, et al. Characterisation of epitopes on barley mild mosaic virus coat protein recognised by a panel of novel monoclonal antibodies. Arch Virol. 2005;150: 2501-2512.

4 Baker JM, Hudson RP, Kanelis V, Choy WY, Thibodeau PH, Thomas PJ, et al. CFTR regulatory region interacts with NBD1 predominantly via multiple transient helices. Nat Struct Mol Biol. 2007;14: 738-745.

5 Hoefer J, Azam MA, Kroetsch JT, Leong-Poi H, Momen MA, Voigtlaender-Bolz J, et al. Sphingosine-1-phosphate-dependent activation of p38 MAPK maintains elevated peripheral resistance in heart failure through increased myogenic vasoconstriction. Circ Res. 2010;107: 923-933.

6 Bui HH, Leohr JK, Kuo MS. Analysis of sphingolipids in extracted human plasma using liquid chromatography electrospray ionization tandem mass spectrometry. Anal Biochem. 2012;423: 187-194.
